# Comparative Transcriptome Analysis Reveals Molecular Insights in Overwintering *Monochamus alternatus* (Coleoptera: Cerambycidae)

**DOI:** 10.1093/jisesa/ieac025

**Published:** 2022-05-13

**Authors:** Hui Li, Xiaohong Xia, Xuanyu He, Shouyin Li, Lulu Dai, Jianren Ye, Dejun Hao

**Affiliations:** 1 Co-Innovation Center for the Sustainable Forestry in Southern China, Nanjing Forestry University, Nanjing, 210037, China; 2 College of Forestry, Nanjing Forestry University, Nanjing, China

**Keywords:** *Monochamus alternatus*, overwintering, transcriptome analysis, immune system, antifreeze compounds

## Abstract

*Monochamus alternatus*, the dominant vector of *Bursaphelenchus xylophilus* (Aphelenchida: Aphelenchoididae), has caused immense damage to forest resources. In China, this vector was native to the southern regions but has spread northward recently. To adapt to more challenging environments in the northern winter, *M. alternatus* has evolved an intricate strategy for overwintering, which remains largely unknown. Herein, we compared the transcriptome data of the overwintering and non-overwintering larvae of *M. alternatus* larvae to investigate the molecular mechanisms in overwintering. A total of 53.10 GB clean bases and 28, 245 unigenes were obtained by RNA-seq. Analysis of 2597 upregulated and 2429 downregulated unigenes, as well as the enrichment of DEGs showed that many genes and pathways were jointly involved in the overwintering period. Besides, the accuracy of the RNA-seq data was tested by using qPCR experiment involving 13 selected genes. The results revealed that the overwintering process relied largely on the energy allocation trade-off. Specifically, overwintering *M. alternatus* inhibited energy-intensive activities, such as growth and molting, detoxification, and trehalose transport, and the reserved energy was skewed towards the synthesis of antifreeze compounds and immune response to cope with the deleterious effects of winter.

The pinewood nematode, *Bursaphelenchus xylophilus* (Aphelenchida: Parasitaphelenchidae), was the pathogen of pine wilt disease (PWD), which was originated from North America (Mamiya and Enda 1972). Since first invaded Nanjing, Jiangsu, China in 1982, PWD has caused immense damage to forest resources and ecosystems in 18 provinces of China (Ye 2019). The pinewood nematode is almost exclusively associated with its symbiont, a pine sawyer beetle *Monochamus alternatus* (Linit 1988, Wang et al. 2014). The geographic distribution of *M. alternatus* partly determines the invasive fate of the nematode population and epidemiology of PWD. In turn, the environmental temperature is a dominant abiotic factor that restrains the insect distribution, particularly by exerting its effect on the course of overwintering (Bale and Hayward 2010).

*Monochamus alternatus* development consists of one or two generations per year in China (Wu et al. 2013). There is evidence that these beetles spend most of their lifetime as larvae in the phloem and xylem of host plants. The fourth- and fifth-instar larvae begin to enlarge the galleries as pupal chambers, which they line with wood shavings for pupation and overwintering (Ma et al. 2006). *Monochamus alternatus* is widely distributed in southern China; however, recently, the range of distribution of this beetle has began to spread northward to high-latitude areas where it is subjected to sub-zero temperatures in winter (Ma et al. 2006, Shi et al. 2019). Long spells of extreme winter conditions easily disrupt the physical barriers of the trunk, which lead to loss of their natural defense barriers. Therefore, a series of intricate strategies for overwintering are pivotal to ensure the survival of individuals and population growth and stability (Shintani 1999).

Overwintering behavior in arthropods is a common strategy to ensure survival to complete life cycles when encountering inevitable extreme temperatures (Teets and Denlinger 2013). Most insects can sense the arrival of winter through changes in the photoperiod and temperature, and adjust their physiological processes in preparation for the extreme cold temperatures (Teets and Denlinger 2013). The insect’s overwintering strategy is generally divided into two distinct categories, namely, freeze tolerance and freeze avoidance (Bale and Hayward 2010). Freeze tolerance species can synthesize ice nucleating agents (proteins) in haemocoel or other ‘safe extracellular areas’ are synthesized to avoid intracellular freezing, whereas all nucleators are removed from the freeze avoidance insects (Lee et al. 1993). Cryoprotectants, such as glycerol, trehalose, and antifreeze protein, are formed in freeze tolerance and freeze avoidance species (Bale and Hayward 2010). Pioneering work on the overwintering strategy of *M. alternatus* indicates that freeze avoidance is dominant in the field (Ma et al. 2006). The overwintering larvae of *M. alternatus* drop supercooling points (SCP) to −15.4°C through acclimation in autumn to avoid freezing and reduces upper limit of cold injury zone (ULCIZ) or increase sum of injurious temperature (SIT) to increase its chilling tolerance (Ma et al. 2006). While these studies have provided information regarding the overwintering process of *M. alternatus*, research into the molecular changes occurring in the overwintering larvae has aided in further elucidation of the underlying mechanisms of survival in severe winter conditions.

RNA-sequencing technology offers an economic and effective method for identification and quantification of potential gene targets, which provides comprehensive information derived from analysis of divergent traits at the transcriptional level. In recent years, with the vigorous development of this approach, research on insect overwintering behavior with respect to molecular aspects has emerged. Studies regarding insect overwintering have also shifted from the determination of SCPs and cold-resistant substances to the expression pattern analysis of targeted proteins and genes in model insects, as well as pests (Misener et al. 2001, Salminen et al. 2015, Durant et al. 2016, Robert et al. 2016, Costa et al. 2020).

Transcriptome analysis of overwintering insects indicates that this physiological process comprises a wide array of interacting genes and pathways. The expression level of genes encoding molecular chaperones, such as heat shock proteins (HSPs), as well as genes involved in cold-protective metabolites synthesis, membrane lipid composition, general cessation of the cell cycle, and immune system response are altered in overwintering insects (Han and Bauce 1998, Michaud and Denlinge 2006, Rinehart et al. 2007). Various insects may differ considerably across stress response mechanisms relevant to overwintering. RNA-seq data of *Anoplophora glabripennis* (Coleoptera: Cerambycidae) in the wintering process have showed that transcripts involved in immune response, including Cathelicidin and serine protease, exhibited significant regulatory roles (Guo 2019). The genes related to metabolic activities, such as the cytochrome oxidase, NADH dehydrogenase, and ATP synthase subunits, are significantly downregulated in *Eogystia hippophaecolus* (Lepidoptera: Cossidae) after low-temperature treatment (Cui et al. 2017). Many insects exhibit an elevation of the transcription of HSPs during the overwintering process (Robert et al. 2016, Yocum et al. 1998, Lopez-Martinez and Denlinger 2008, Xia et al. 2013, Kang et al. 2016, Tusong et al. 2017).

In the present study, the cDNA libraries of overwintering *M. alternatus* larvae were constructed. Subsequently, we focused on comparative analysis of differential expression of genes and pathways between overwintering and non-overwintering larvae together with qPCR tests to validate the correction of RNA-seq data. Our study enriches the current *M. alternatus* transcriptome database and elucidates intrinsic events of its overwintering behavior, which allows further exploration function of overwintering-related genes to understand the wide spread of *M. alternatus* to temperate regions.

## Materials and Methods

### Insects Collection

*Monochamus alternatus* larvae were originally collected from Masson pine forests of Lushan mountain range in Jiujiang city, Jiangxi province, China (N 29°45ʹ, E 116°05ʹ). *Monochamus alternatus* includes one generation and 4th-instar larvae (larval stage was divided into five instars) came into the overwintering form from November to February at the sampling site.

**Fig. 1.**
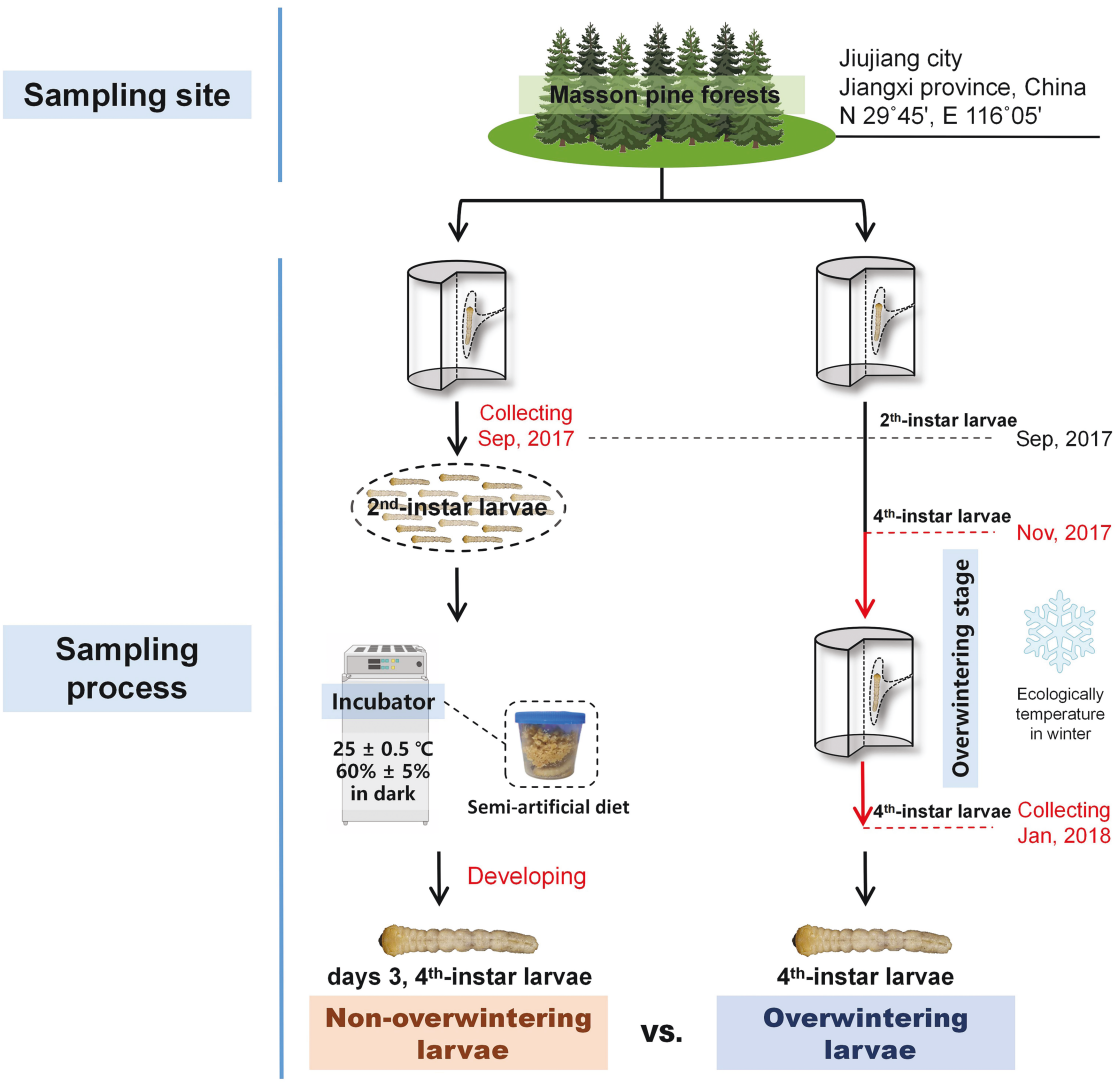
Procedure for sampling of non-overwintering and overwintering groups.

The scheme for insects sampling is shown in Fig. 1. To obtain fourth-instar non-overwintering larvae, the second-instar larvae were collected from three infested trunks of Masson pine at the field in September 2017. These larvae were reared individually on the semi-artificial diet (wood chips of Masson pine as the principal constituent) in an incubator at a constant temperature of 25 ± 0.5°C (RH: 60% ± 5%; in dark). Nine days 3, fourth-instar larvae reared in the laboratory (three individuals as a sample pooling) had been sampled as non-overwintering larvae in our previous study (Li et al. 2020). The overwintering fourth-instar larvae were directly collected from another three infested trunks close to the trunks of non-overwintering larvae collections in January 2018 (monthly mean maximum temperature: 6°C; monthly mean minimum temperature: 0°C). Nine fourth-instar overwintering larvae (three individuals as a sample pooling) were sampling as overwintering larvae. All samples were immediately frozen in liquid nitrogen and stored at −80°C until RNA extraction.

### RNA-Seq Library Construction and Sequencing

Each sample pooling of overwintering larvae was separately crushed in liquid nitrogen, and 100 mg obtained powder were instantly transferred to 2-ml RNase-free centrifuge tubes for RNA extraction using Trizol Reagent (Tiangen, Beijing, China) according to the manufacturer’s protocol. The quantity and purity of the total RNA were examined by separating RNA samples on 1% agarose gels using the NanoDrop 2000 (Termo, Waltham, MA) and Agilent 2000 (Agilent, San Diego, CA). Three biological replicates of overwintering larvae were used for cDNA library construction. The complementary DNA (cDNA) libraries were generated with NEBNext UltraTM RNA Library Prep Kits for Illumina (NEB, Beverly, MA) following the manufacturer’s protocol. Briefly, after isolation by Oligo(dt) magnetic beads, mRNA was fragmented randomly using divalent cations under elevated temperature in NEBNext First Strand Synthesis Reaction Buffer and approximately 300 bp fragments were obtained. Thereafter, based on the mRNA fragment, the stable double-strand cDNA (ds cDNA) was synthesized by using random hexamers and reverse transcriptase. Adenine and sequencing adaptors were ligated at the 3ʹ-end of purified dscDNA followed by PCR reactions for construction of a cDNA library. Next, sequencing was performed using the Illumina TruseqTM RNA sample prep Kit on Illumina Novaseq 6000 with the paired-end read module (Shanghai Majorbio Bio-pharm Biotechnology Co., Shanghai, China). RNA-Seq raw data of overwintering larvae were deposited in NCBI Sequence Read Archive (NCBISRA) database with accession numbers SRX8715591, SRX8715592, and SRX8715593.

cDNA library of non-overwintering larvae had been constructed and sequenced in our previous study following the above method (Li et al. 2020). RNA-Seq raw data of non-overwintering larvae were deposited on Genebank database with accession numbers SRX6034733, SRX6034734, and SRX6034736.

### Assembly and Functional Annotation

Raw reads were filtered by removing reads containing adapters and >10% ambiguous ‘N’ nucleotides and low-quality reads (defined as reads with >20% of the bases having quality scores < 10) in SeqPrep software (https://github.com/jstjohn/SeqPrep) before transcriptome assembly. The Q20, Q30, and GC contents of the cleaned datasets were calculated to assess sequencing quality in fastx_toolkit_0.0.14 software (http://hannonlab.cshl.edu/fastx_toolkit/). Quantified clean reads were assembled without a reference genome by using TRINITY with default parameters (Grabherr et al. 2011). The nonredundant sequences were obtained by removing sequences showing redundancy in CD-HIT software (http://weizhongli-lab.org/cd-hit/), and the longest sequence of each cluster was assigned to one unigene.

To annotate the obtained unigenes, NCBI nonredundant proteins (NR) (https://ftp.ncbi.nlm.nih.gov/blast/db/), Swiss-Prot (www.ebi.ac.uk/uniprot/), and Clusters of Orthologous Groups (COG) (www.ncbi.nlm.nih.gov/COG/) databases were searched using DIAMOND software (version 0.8.37.99) with a cut-off value of 1e^−5^. Protein families (Pfam) (http://pfam.xfam.org/) database was searched using HMMER3 software (version 3.1b2) with default parameters. Gene Ontology (GO) database (www.geneontology.org/) was searched using BLAST2GO software (version 2.5.0) with default parameters. Kyoto Encyclopedia of Genes and Genomes (KEGG) database (www.genome.jp/kegg/) was searched using KOBAs software (version 2.1.1) with default parameters. In addition, NR annotation provided the information on the species distribution of the top BLASTX hits. Based on GO and KEGG annotations, the annotated unigenes were further classified into different functional groups (i.e., GO terms and KEGG pathways).

### Differential Gene Expression Analysis

We used Fragments Per Kilobase per Million (FPKM) reads by Cuffdiff software (version 2.1.0) (http://cole-trapnell-lab.github.io/cufflinks/) to represent the gene expression levels. Differential expression analysis was conducted by using the DESeq2 software (Andes and Huber 2010). Fold change (FC) values for gene expression were considered significant if *P*-value < 0.05 (false discovery rate ≤ 0.01) and |log2FC| ≥ 1. Differential expression levels of genes were then further utilized for GO and KEGG enrichment analyses. After the *P*-value of < 0.05 was calculated by the Fisher’s test in the enrichment analysis, GO and KEGG enrichment were analyzed by using the Goatools (https://github.com/tanghaibao/GOatools) and Perl script software, respectively (Liu et al. 2014). All the heatmaps of differential expression genes were with OriginPro software (OriginLab Inc., Northampton, United Kindom).

### Quantitative PCR Validation

Initially, five upregulated and eight downregulated unigenes were selected to support the accuracy of the RNA-seq results. Total RNA of non-overwintering and overwintering samples were extracted using the methods described above. cDNA was synthesized using 1 μg total RNA template following the instructions outlined by the 1st Strand cDNA Synthesis Kit (Vazyme, Nanjing, China). The expression levels of 13 genes were determined by qPCR using the Hieff UNICON qPCR SYBR Green Master in the Applied Biosystems 7500 System (USA) according to the manufacturer’s instructions. The PCR procedure was as follows: 5 min at 95°C, 40 cycles of 10 s at 95°C, and 40 s at 60°C, followed by melting curve analysis. Sequence and efficiency of the primer is provided in Supp Table S1 (online only). Relative expression levels were measured based on the 2^−ΔΔCt^ method with Ribosomal Protein 10 (RPL10) as the internal control for the normalization of data (Li et al. 2018). To verify the reliability of RNA-Seq data, Pearson’s correlation between fold changes (Log2 transformed) in RT-qPCR and RNA-Seq results was analyzed.

## Results

### Sequencing, Assembly, and Annotation

**Fig. 2.**
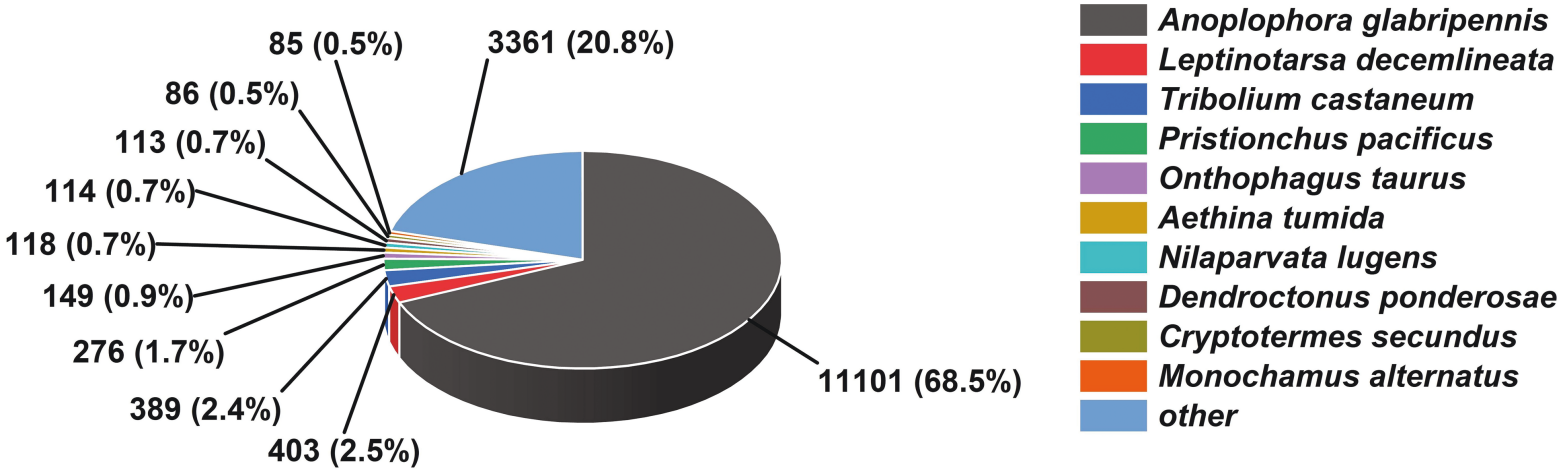
Species distribution of BLASTx matches the unigenes of *M. alternatus* non-overwintering and overwintering larvae. Each piece of fan indicates the number and ratio of the top BLASTx matches against the Genebank non-redundant (NR) database for various species.

We performed transcriptome sequencing using larvae of *M. alternatus* in non-overwintering and overwintering conditions, and obtained approximately 53.10 GB of clean bases, 321,155,974 raw reads, and 311,027,396 clean reads after adaptor trimming and quality filtering. These clean reads were assembled into 28,245 unigenes with an average length of 1165 bp, N50 of 1762 bp, E90N50 of 2125 bp, 38.60% GC content, and over 93% clean reads ratio (Table 1). Supp Fig. S1 (online only) showed that the lengths of 64% (18,056) of the total unigenes exceeded 500bp and the lengths of 48% (10,606) of the total unigenes exceeded 1,000bp. The six databases described above were utilized to perform gene annotation. Annotation showed that most unigenes (57.22%) were mapped into the NR database, 44.28%, and 47.94% of these unigenes were annotated with Swiss-Prot and Pfam database individually (Table 2). In contrast, in the species distribution, *M. alternatus* sequences showed high matches (68.91%) with *A. glabripennis* (Fig. 2), followed by *Leptinotarsa decemlineata* (Coleoptera: Chrysomelinae) (2.50%) and *Tribolium castaneum* (Coleoptera: Tenebrionidae) (2.41%). Moreover, we detected 33,060 coding sequences by performing functional annotations.

**Table 1.** Summary of RNA-seq data in *M. alternatus*

Summary of RNA-seq data	
Total number of raw reads	321,155,974
Total number of clean reads	311,027,396
Total number of clean bases	53,103,958,608
Total number of unigenes	28,245
Total sequence base (bp)	45,332,941
Average length (bp)	1165.64
N50	1762
E90N50	2125
GC(%)	38.60
Clean reads Q20 (%)	97.74
Clean reads Q30 (%)	93.55

**Table 2.** Summary of the annotation of the assembled RNA-seq data

Database	Unigenes number	Percentage(%)
NR	16162	57.22
Swiss-Prot	12507	44.28
Pfam	13542	47.94
COG	3152	11.16
GO	9092	32.19
KEGG	9412	33.32
Total unigenes	28245	100

### GO and KEGG Analyses

**Fig. 3.**
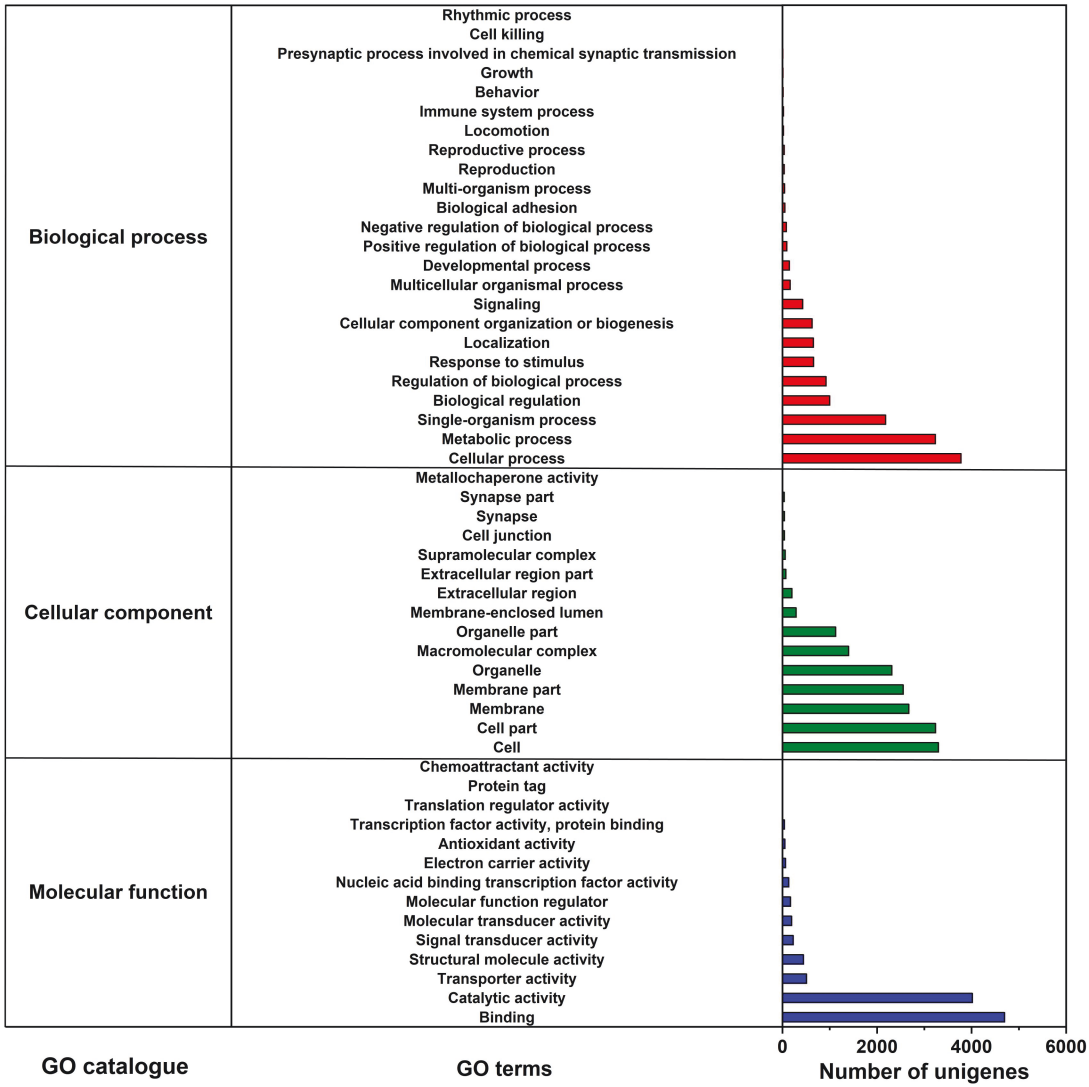
Summary of GO analysis of the unigene sequences of *M. alternatus* non-overwintering and overwintering larvae. The x-axis on the right indicates the number of unigenes in a category.

**Fig. 4.**
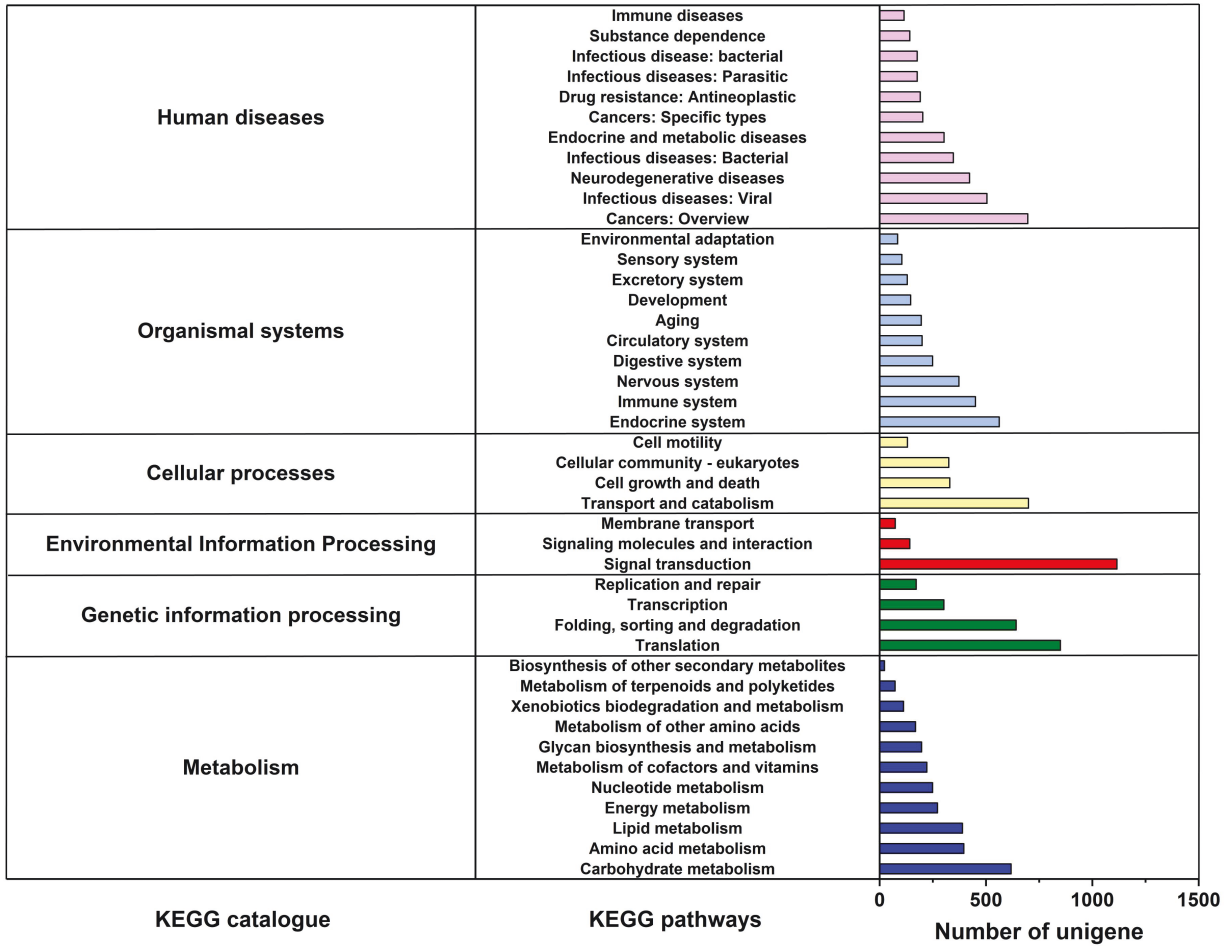
KEGG metabolic pathway of *M. alternatus* non-overwintering and overwintering larvae. The x-axis on the right indicates the number of unigenes in a category.

We categorized unigenes into three categories (biological processes, cellular components, and molecular function) and 53 subcategories by GO analysis. Among these, ‘cellular process’ (26.64%) and ‘metabolic process’ (22.82%) were dominant terms in the ‘biological process’. ‘cell part’ (18.67%) and ‘cell’ (19.03%) terms were abundant mostly in the ‘cellular components’ items. Within the ‘molecular function’ category, unigenes were mostly assigned with ‘binding’ (44.54%) and ‘catalytic activity’ (38.09%) subcategories (Fig. 3). Additionally, 13,285 unigenes were mapped to 43 KEGG secondary pathways in this study. ‘Folding, sorting and degradation’, ‘translation’, ‘carbohydrate metabolism’, ‘transport and catabolism’, and ‘nervous system’ were the main secondary pathways, except those related to human diseases (Fig. 4).

### DEG Functional Enrichment Analysis

Further examination of fold-change differences showed that 5026 unigenes were differentially expressed genes (DEGs) between non-overwintering and overwintering larvae *M. alternatus* (*P*-value < 0.05, |log2FC| ≥ 1). Among all DEGs identified from overwintering and non-overwintering larvae datasets, 2125 and 2139 genes were respectively mapped to 251 and 299 terms in GO and KEGG enrichment. Additionally, we eliminated data on pathways related to human diseases. The top 10 significantly enriched GO terms of DEGs between non-overwintering and overwintering larvae are listed in Table 3. Most enriched GO terms were involved in ‘cellular processes and components’, which included the category of ‘organic and hetero cyclic compound binding’, ‘integral and intrinsic components of the membrane’, and so on. The ‘catalytic activity’ and ‘metabolic process’ were also enriched (Table 3).

**Table 3.** The top 10 significantly enriched GO terms between non-overwintering larvae and overwintering larvae

GO ID	Description	DEG numbers	Ratio	*P*
GO:0008150	Biological process	997	0.47	0.003
GO:0003824	Catalytic activity	854	0.40	0.007
GO:0097159	Organic cyclic compound binding	690	0.32	0.004
GO:1901363	Hetero cyclic compound binding	689	0.32	0.004
GO:0044425	Membrane part	659	0.31	0.048
GO:0044464	Cell part	656	0.30	0.003
GO:0009987	Cellular process	640	0.30	0.003
GO:0016021	Integral component of membrane	623	0.29	0.009
GO:0031224	Intrinsic component of membrane	623	0.29	0.010
GO:0008152	Metabolic process	601	0.28	0.003

The top 10 significantly enriched KEGG terms of DEGs between non-overwintering and overwintering larvae are listed in Table 4. KEGG pathway enrichment showed that 35 and 23 DEGs were respectively mapped to the ‘longevity regulating pathway’, as well as ‘worm and insect hormone biosynthesis categories’. The other enriched KEGG pathways were mostly correlated with a wide array of metabolic process, such as the metabolism of amino acid, glucose, and toxic substances. Besides, 32 DEGs, including unigens encoding Cadherin, Actin, and Pkinase, were enriched in ‘Hippo signaling pathways’. Notably, in the enrichment of the ‘longevity regulating pathway’, nine small Heat Shock Protein (sHSP) genes were upregulated in the overwintering larvae (Supp Fig. S2 [online only]). In the enrichment of the ‘worm and insect hormone biosynthesis categories’, two unigenes encoding Aldehyde dehydrogenase (ALDH) and nine unigenes encoding Short chain dehydrogenase were downregulated in the non-overwintering larvae (Supp Fig. S3 [online only]).

**Table 4.** The top 10 significantly enriched KEGG pathways except those related to human diseases between non-overwintering larvae and overwintering larvae of *M. alternatus* unigenes

Pathway ID	Description	DEG numbers	Ratio	*P*
map04212	Longevity regulating pathway—worm	35	0.016	0.013
map04391	Hippo signaling pathway-fly	31	0.014	< 0.001
map00980	Metabolism of xenobiotics by cytochrome P450	31	0.014	0.003
map04390	Hippo signaling pathway	30	0.014	0.016
map00310	Lysine degradation	29	0.014	0.002
map00040	Pentose and glucuronate interconversions	29	0.014	0.018
map00982	Drug metabolism—cytochrome P450	28	0.013	0.005
map00480	Glutathione metabolism	26	0.012	0.028
map00053	Ascorbate and aldarate metabolism	25	0.012	0.269
map00981	Insect hormone biosynthesis	23	0.011	0.005

### Gene Expression Analysis

**Fig. 5.**
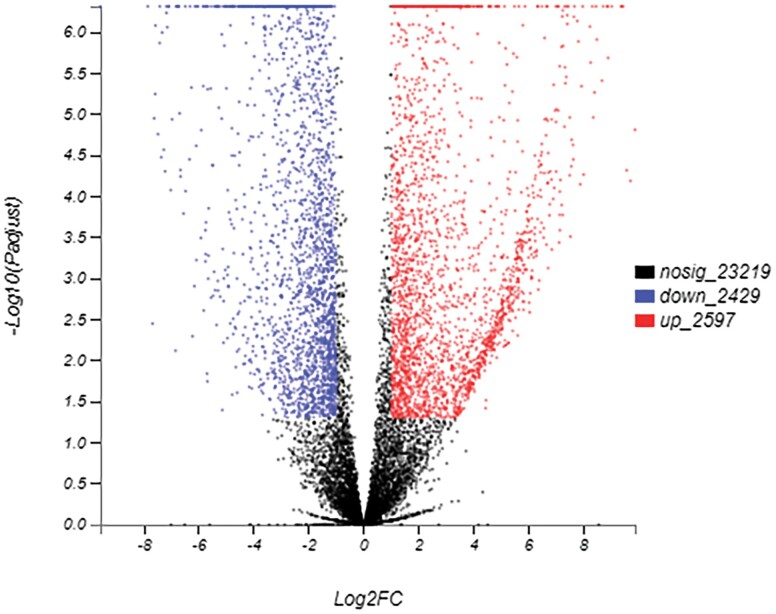
Differentially expressed genes (DEGs, *P*-value < 0.05 and |log2FC| ≥ 1) in *M. alternatus* between non-overwintering larvae and overwintering larvae. Scatter plot of DEGs illustrating the full set of genes. Red points are up-regulated genes, blue points are down-regulated genes, and black points are non-DEGs.

**Fig. 6.**
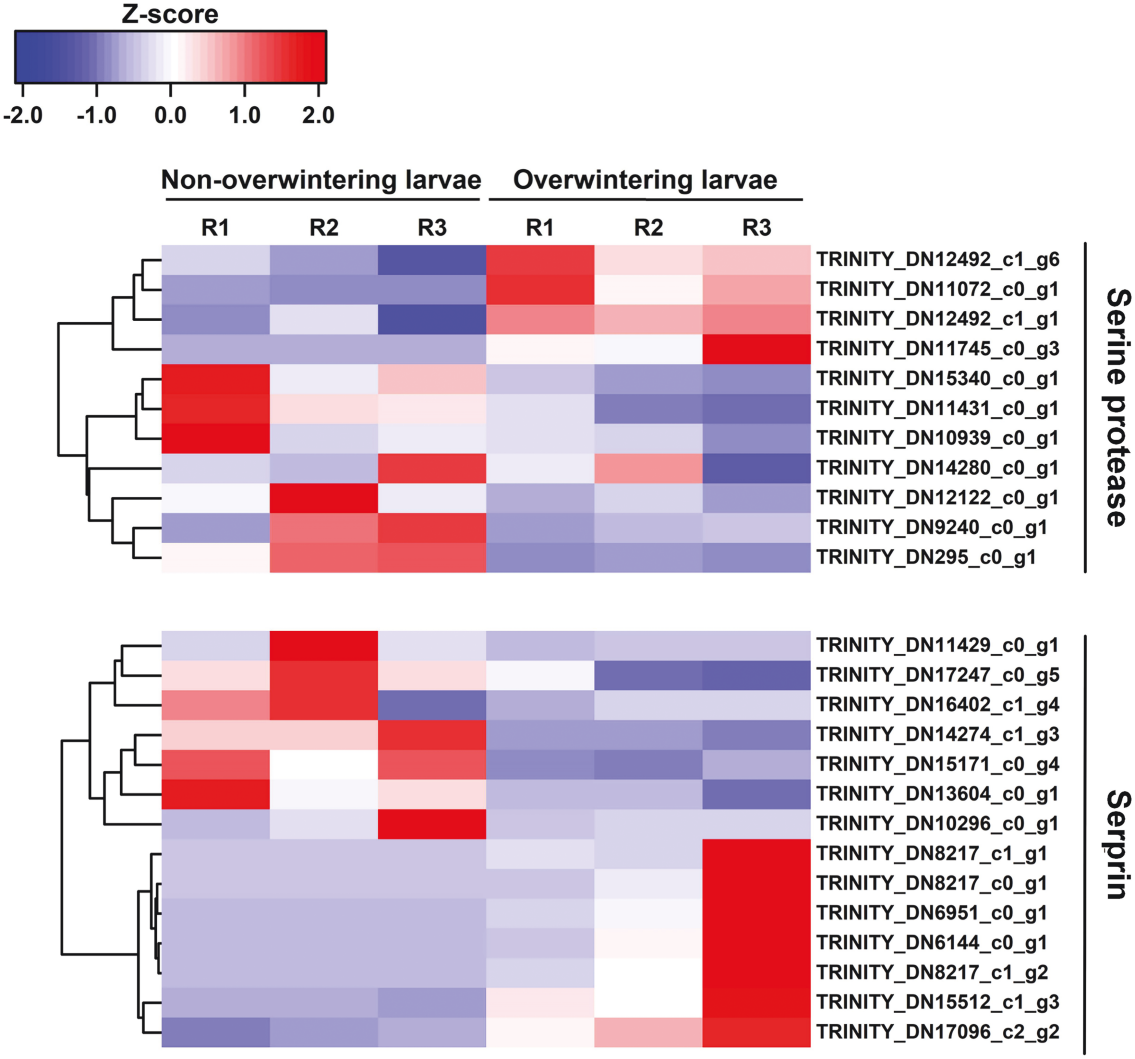
Heatmap and cluster analysis of DEGs encoding Serine protease and Serprin in non-overwintering larvae and overwintering larvae. The color scale bar showed expression levels after Z-score row normalization. Red bar indicated up-regulated, whereas blue bar indicated down-regulated. R1-3 represented three replicates.

Overall, 2597 and 2429 unigenes were upregulated and downregulated respectively in overwintering larvae (Fig. 5). The 15 most upregulated and 12 most downregulated genes are listed in Table 5. Many unigenes related to immune response were identified among the most upregulated genes, including the serine protease inhibitor (Serpin), arrestin homolog, leukocyte elastase inhibitor, and early endosome antigen. Two unigenes encoded cytochrome P450 and one PBAN-type neuropeptide gene was significantly induced. We performed further heatmap analysis of the serine protease and serpin, the dominant component of the immune system. As shown in Fig. 6, expression levels of four of the eleven serine proteases and half of the serpin unigenes were elevated in the overwintering larvae.

**Table 5.** Differentially expressed genes between non-overwintering larvae and overwintering larvae

	Gene ID	*P*	Annotation (Subject)	Log 2 (Fold change) overwintering larvae /non-overwintering larvae	GenBank No.
Up- regulated genes	TRINITY_ DN8217_c1_g2	< 0.001	Serpin (*Pieris rapae*)	9.39	OM349508
	TRINITY_ DN11745_c0_g3	< 0.001	Putative serine protease (*Anoplophora glabripennis*)	9.07	OM349509
	TRINITY_ DN12388_c0_g1	< 0.001	Cytochrome P450 (*Anoplophora glabripennis*)	9.04	OM349510
	TRINITY_ DN9328_c0_g1	< 0.001	Zinc finger MYM-type protein 1-like (*Vollenhovia emeryi*)	7.66	OM349511
	TRINITY_ DN16720_c0_g2	< 0.001	Trypsin (*Anoplophora glabripennis*)	7.41	OM349512
	TRINITY_ DN10090_c0_g2	< 0.001	Arrestin homolog (*Anoplophora glabripennis*)	7.02	OM349513
	TRINITY_ DN8907_c0_g1	< 0.001	Late embryogenesis abundant protein (*Polypedilum vanderplanki*)	7.01	OM349514
	TRINITY_ DN8217_c0_g1 TRINITY_ DN7374_c0_g1 TRINITY_ DN17361_c1_g2 TRINITY_ DN17350_c4_g3 TRINITY_ DN370_c0_g1 TRINITY_ DN13471_c1_g4 TRINITY_ DN14283_c0_g1 TRINITY_ DN8397_c0_g1	< 0.001 < 0.001 < 0.001 < 0.001 < 0.001 < 0.001 < 0.001 < 0.001	leukocyte elastase inhibitor-like isoform X3 (*Nilaparvata lugens*) odorant-binding protein 19 (*Monochamus alternatus*) golgin subfamily A member 5-like (*Anoplophora glabripennis*) Transaldolase (*Anoplophora glabripennis*) PBAN-type neuropeptides-like (*Anoplophora glabripennis*) ATP synthase subunit beta, mitochondrial (*Anoplophora glabripennis*) cytochrome P450 4d14 (*Anoplophora glabripennis*) early endosome antigen 1-like (*Anoplophora glabripennis*)	6.86 6.65 6.58 6.33 5.83 5.80 5.78 5.72	OM349515 OM349516 OM349517 OM349518 OM349519 OM349520 OM349521 OM349522
Down- regulated genes	TRINITY_ DN9677_c0_g1	< 0.001	Catalase-like (*Anoplophora glabripennis*)	−9.59	OM349523
	TRINITY_ DN17927_c0_g1	< 0.001	Sorbitol dehydrogenase (*Daphnia magna*)	−7.62	OM349524
	TRINITY_ DN13804_c0_g4	< 0.001	Cytochrome C peroxidase, mitochondrial (*Orchesella cincta*)	−7.38	OM349525
	TRINITY_ DN9551_c0_g1	< 0.001	Facilitated trehalose transporter (*Anoplophora glabripennis*)	−7.43	OM349526
	TRINITY_ DN19812_c0_g1	< 0.001	Endocuticle structural glycoprotein (*Anoplophora glabripennis*)	−6.68	OM349527
	TRINITY_ DN13783_c1_g1	< 0.001	Glucose dehydrogenase (*Anoplophora glabripennis*)	−6.52	OM349528
	TRINITY_ DN16543_c0_g1	< 0.001	Chitinase (*Monochamus alternatus*)	−6.03	OM349529
	TRINITY_ DN295_c0_g1	< 0.001	Serine protease (*Anoplophora glabripennis*)	−5.94	OM349530
	TRINITY_ DN21452_c0_g1	<0.001	Pupal cuticle protein (*Anoplophora glabripennis*)	−5.90	OM349531
	TRINITY_ DN14499_c0_g3	< 0.001	Chloride channel protein 2 (*Anoplophora glabripennis*)	−5.84	OM349532
	TRINITY_ DN13740_c0_g9	< 0.001	Aminopeptidase N (*Anoplophora glabripennis*)	−5.72	OM349533
	TRINITY_ DN14375_c0_g4	< 0.001	UDP-glucuronosyl transferase (*Anoplophora glabripennis*)	−5.66	OM349534

**Fig. 7.**
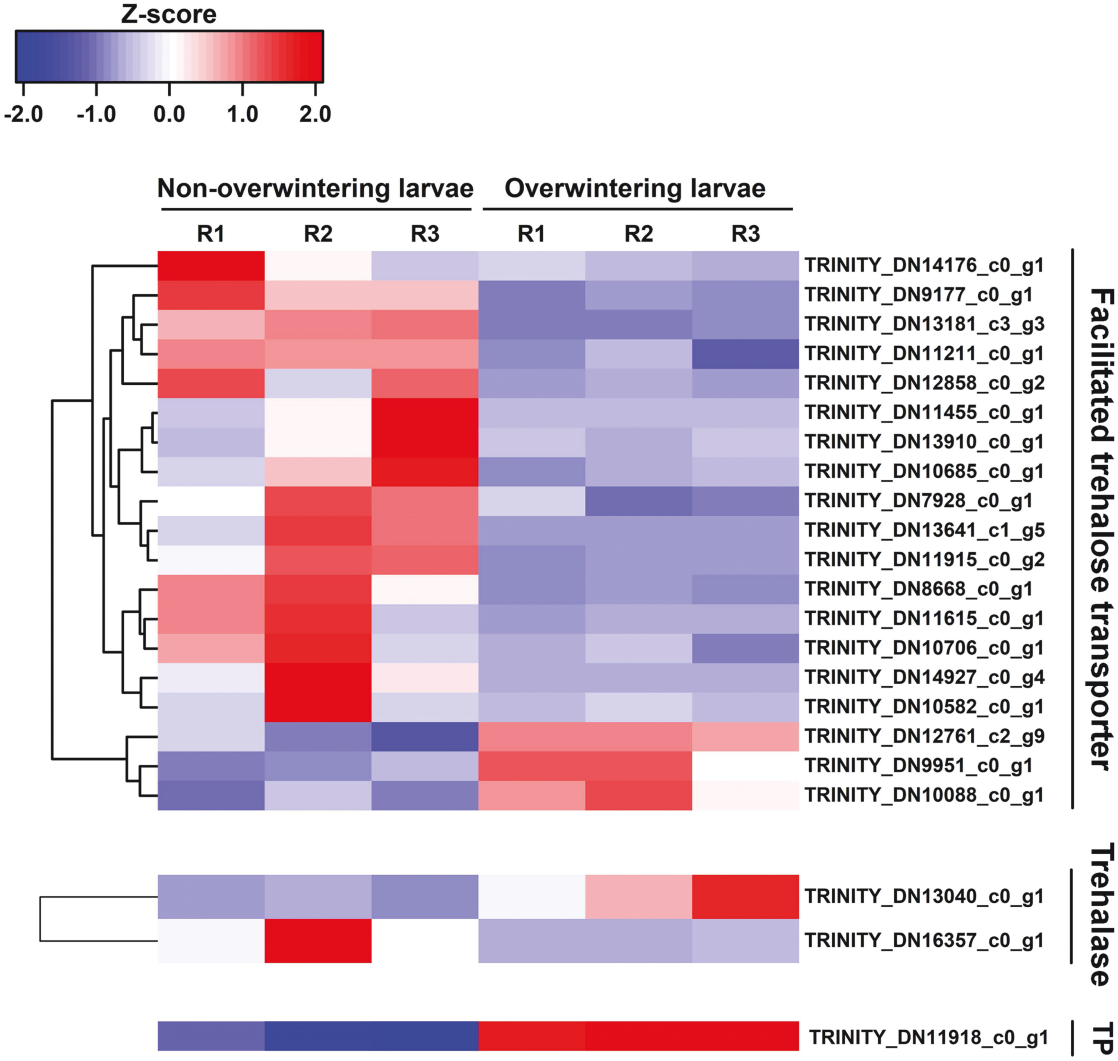
Heatmap and cluster analysis of DEGs encoding facilitated trehalose transporter, Trehalase, and Trehalase phosphatase (TP) in non-overwintering larvae and overwintering larvae. The color scale bar showed expression levels after Z-score row normalization. Red bar indicated up-regulated, whereas blue bar indicated down-regulated. R1-3 represented three replicates.

As shown in Table 5, in the overwintering larvae, a wide array of unigenes involved in antioxidant and metabolic reactions were inhibited, including catalase-like, cytochrome C peroxidase, mitochondria, UDP-glucuronosyltransferase, sorbitol dehydrogenase, and glucose dehydrogenase. Additionally, several abundant downregulated genes including those for endocuticle structural glycoprotein, chitinase, and pupal cuticle protein were contributors to the insect metamorphosis process. Overall, 24 genes related to trehalose were assessed in this study, and 19 of the 21 genes encoding facilitated trehalose transporter (TRET) were inhibited during this process (Fig. 7). Moreover, one gene annotating trehalose phosphatase and one gene encoding trehalase were induced in overwintering larvae (Fig. 7).

### qRT-PCR Validation

**Fig. 8.**
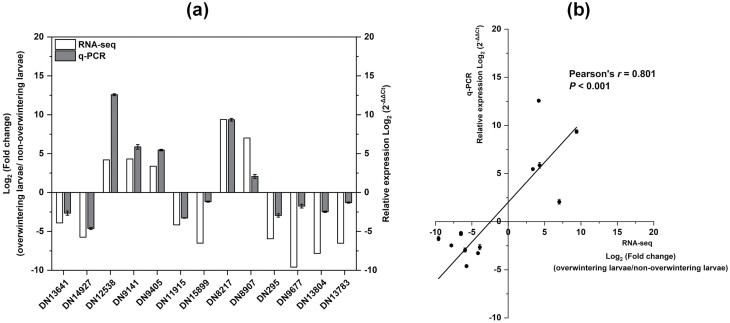
Validation of DEGs by qRT-PCR. (a) Relative expression levels of thirteen DEGs via RNA-seq and qRT-PCR. DN1364, facilitated trehalose transporter; DN14927, facilitated trehalose transporter; DN12538, heat shock protein 20; DN9141, heat shock protein 23; DN9405, heat shock protein 20; DN11915, facilitated trehalose transporter; DN15899, endocuticle structural glycoprotein; DN8217, serpin; DN8907, late embryogenesis abundant protein; DN295, serine protease; DN9677, catalase; DN13804, cytochrome c peroxidase; DN13783, glucose dehydrogenase. Ribosomal protein10 was used as the internal control. (b) Correlation between RNA-Seq and qRT-PCR results for the tested DEGs using Pearson correlation coefficient (*P* < 0.05).

The expression levels of many transcripts changed in overwintering larvae. qRT-PCR performed for 13 randomly selected genes supported the accuracy of RNA-seq (Fig. 8). Five unigenes, including those encoding HSP20 (DN12538, DN9141, and DN9405), leukocyte elastase inhibitor (DN8217), and late embryogenesis abundant protein (DN8907), were upregulated, and the other eight unigenes, including those encoding TRET (DN1364, DN14927, and DN11915), Endocuticle structural glycoprotein (DN15899), Serine protease (DN295), Catalase (DN9677), Cytochrome c peroxidase (DN13804), and Glucose dehydrogenase (DN13783), were downregulated (Fig. 8a). In addition, the qRT-PCR results were highly consistent with RNA-seq results (Pearson’s *r* = 0.801, *P* < 0.001; Fig. 8b).

## Discussion

RNA-seq technology has been widely used in *M. alternatus* to investigate molecular events in various physiological processes, exemplified by the responses to pesticides and high temperatures (Li et al. 2020, Livak and Schmittgen 2001). Here, we compared the transcriptome data of *M. alternatus* in overwintering and non-overwintering larvae to explore the mechanisms by which this beetle coped with cold winters at the molecular level. Total of 53.10 GB clean bases and 28,245 unigenes were observed in this study, which substantially enriched the existing *M. alternatus* transcriptome database. Similar to the findings obtained in the previous study, high matches with the transcriptome database of *A. glabripennis* accurately reflected the evolutionary process (Li et al. 2020).

Most highly annotated GO terms of *M. alternatus* transcriptome were strikingly conserved in various experimental treatments. Lin et al (2015) performed RNA-sequencing on *M. alternatus* (pooling samples of larvae, pupae and adults reared under laboratory conditions) and found that the most abundant GO terms were the ‘cellular process’, ‘cell’, ‘cell part’, ‘binding’ and ‘catalytic activity’. The same highly annotated GO terms were presented by not only the tanscriptome of *M. alternatus* non-overwintering and overwintering larvae in this study, but also the tanscriptome of *M. alternatus* larvae submitted to heat exposure in our previous study (Li et al. 2020). In contrast, the KEGG analysis results differed largely across different treatments. The dominant KEGG pathway of *M. alternatus* under laboratory conditions included ‘metabolic pathways’, whereas those related to ‘environmental information processing’ were abundant in the overwintering larvae (Wu et al. 2016, Lin et al. 2015). This indicated that the overwintering process was accompanied by a wide array of intricately physiological, biochemical, and molecular changes to *M. alternatus*, which was pivotal for distribution in the zones where the mean air temperature isotherm is above −10°C (Ma et al. 2006).

Overall, 5026 DEGs were observed in *M. alternatus* overwintering larvae, and the most upregulated and downregulated genes are listed in Table 5. Our qPCR study supports the accuracy of DEG analysis. Overwintering was essentially a process of achieving efficient energy utilization, involving complicated energy trade-off between basal metabolism activities and stress response (Sinclair et al. 2013, Sinclair 2015).

### Inhibition of Genes and Pathways Involved in Energy-Intensive Physiological Activities

The results of GO and KEGG enrichment analyses showed that many DEGs between *M. alternatus* non-overwintering and overwintering larvae were mapped to ‘Catalytic activity’ and ‘Metabolic process’ terms. Further identification of DEGs found that unigenes encoding mitochondria, sorbitol dehydrogenase, and glucose dehydrogenase were inhibited in *M. alternatus* overwintering larvae, which reflected the low metabolic rates during overwintering. No feeding behavior occurred in overwintering larvae, which mean that *M. alternatus* larvae did not need to cope with the secondary metabolism product from host plants (Feyereisen 1999, Pan et al. 2020). Thus, in overwintering *M. alternatus* larvae, some downregulated genes concentrated on the group of enzymes commonly associated with the detoxification of xenobiotics.

Another group of enriched DEGs involved transcripts related to trehaloses. Trehalose was the main sugar component of insect hemolymph, which was synthesized in the fat body, released into the hemolymph, and subjected to uptake by other tissues (Thompson 2003, Wyatt 1967). Facilitated trehalose transporter (TRET) can disrupt the impermeability of cellular membranes, a major obstacle to trehalose (Kikawada et al. 2007). The result was that 19 TRET genes were inhibited in overwintering *M. alternatus*, which hinted at the low exchange rate of trehalose in winter, consistent with the slow metabolism described above.

The genes and pathways involved in development and molting were inhibited in overwintering *M. alternatus* larvae. Chitinases and endocuticle structural glycoprotein were essential for insect molting and development (Arakane and Muthukrishnan 2010). Moreover, pupal cuticle proteins were synthesized by the imaginal disk epithelium before pupation (Nakato 1990). The inhibition of these unigenes in *M. alternatus* overwintering larvae confirmed the fact that the growth and development were arrested in the process of overwintering (Palli 2001). In contrast, the non-overwintering larvae reared at constant temperatures undergo molts and pupate directly without overwintering (Chen et al. 2017). In the overwintering *Microdera punctipennis* (Coleoptera: Tenebrionidae), DEGs were enriched in ‘insect hormone biosynthesis’ pathway (Tusong et al. 2017), which showed consistency with our results. Further identification of DEGs in this pathway observed that unigenes encoding Aldehyde dehydrogenase (ALDH) and short-chain dehydrogenase were downregulated *M. alternatus* overwintering larvae. Both the ALDH and short-chain dehydrogenase contributed to biosynthesis of Juvenile hormone and Molting hormone (Kavanagh et al. 2008, Rivera-Perez et al. 2013). Inhibition of these genes in *M. alternatus* overwintering larvae suggested that the failure to molt during overwintering might be attributed to the shutdown of ecdysteroids.

Diapause, a genetically determined hormone-mediated state of inhibited development, was a vital strategy for insects to cope with unfavorable environmental conditions, such as low temperatures (Palli et al. 2001). PBAN, a major family of neuropeptides in insects, participated in the induction and termination of diapause (Choi et al. 2013). Previous studies argued that *M. alternatus* larvae exhibited facultative diapause (Togashi 2019). One Pheromone Biosynthesis Activating Neuropeptide (PBAN) unigene was included among the top upregulated genes in overwintering larvae, supporting the diapause behavior of *M. alternatus* (Togashi 2019).

### Induction of Genes and Pathways Involved in Stress Response During Overwintering

Insects could elevate the synthesis of antifreeze compounds to cope with winter. Trehalose was a common cryoprotectant in insects, which might compensate for osmotic shock and stabilize cellular membrane structures under low temperatures (Storey and Storey 1991). Early investigations found that the level of trehalose was elevated in overwintering insects, and with the widespread application of sequencing technology, studies in *Cydia pomonella* (Lepidoptera: Tortricidae), *Drosophila* (Diptera: Drosophilidae) species, and *L. decemlineata* have found that the expression levels of genes associated with trehalose are synchronously upregulated (Rozsypal et al 2013, Olsson et al. 2016, Govaere et al. 2019). The elevated expression levels of trehalose phosphatase and trehalase unigenes indicated that trehalose might be the critical cryoprotectants in *M. alternatus*.

Small heat shock proteins (sHSPs), involved in longevity regulating pathway category, were another class of protective regents contributing to the cold tolerance of overwintering insects. Dietary restriction (DR) inhibited the PI3K/Akt/TOR intracellular signaling cascade and consequently activated the antiaging FOXO family transcription factors, which induced the transcription of genes related to resistance, including sHSPs (Mair and Dillin 2008, Kalaany and Sabatini 2009). sHSP, belonging to the Heat Shock Protiens (HSPs) superfamily, was originally described in *Drosophila melanogaster* (Diptera: Drosophilidae) in response to heat stress (Feder and Hofmann 1999, Crack et al. 2002, Wang et al. 2012, Zhang et al. 2014). The elevation of sHSP during the overwintering process has been documented in many insects, such as *Dendroctonus ponderosae* (Coleoptera: Scolytidae) (Robert et al. 2016), *Sarcophaga crassipalpis* (Diptera: Sarcophagidae) (Yocum et al. 1998), *Rhagoletis pomonella* (Diptera: Tephritidae) (Lopez-Martinez and Denlinger 2008), *Plutella xylostella* (Lepidoptera: Plutellidae) (Xia et al. 2013), *Culex pipiens* (Diptera: Culicidae) (Kang et al. 2016), *M. punctipennis* (Tusong et al. 2017), and *Hyphantria cunea* (Lepidoptera: Erebidae) (Deng et al. 2018). Inhibition of the expression of HSP23 in *S.crassipalpis* by RNAi exerted a negative effect on pupal defense mechanisms against low temperatures (Rinehart 2007). Expression levels of nine sHSPs were upregulated in the overwintering *M. alternatus* larvae, which confirmed that sHSP was critical to the cold tolerance during insect overwintering. The occurrence of DR in overwintering *M. alternatus* might activate the longevity regulating pathway and lead to the upregulation of sHSPs to stabilize the protein structure or to relieve damage in the course of adaptation against deleterious effects of winter.

Except for synthesis of antifreeze compounds, the immune system was activated during insect overwintering since it was necessary for most insects to counter pathogens that cause overwintering-associated mortality. Previous discoveries have also stated that defensin proteins related to the immune response may serve as protectants against freezing (Palli et al. 2001, Ferguson et al. 2016, Salehipour-shirazi et al. 2017). Therefore, it was not surprising that some unigenes associated with the immune response were upregulated in overwintering *M. alternatus* larvae, and similar results have been observed in *S.crassipalpis*, *Caenorhabditis elegans* (Rhabditia: Rhabditidae), *D. melanogaster*, *Megachile rotundata* (Hymenoptera: Megachilidae) (Ragland et al. 2020; Xu and James 2012). Eleven serine proteases and twelve serpins were observed from the DEGs, and two serine proteases and one serpins were identified among the top fifteen DEGs. Serine proteases play critical roles in a variety of insect immune responses and were irreversibly inactivated by serine protease inhibitors (serpins) to perform regulatory functions (Gorman et al. 2000). The serpin-serine protease pairs regulated innate immune responses represented by the Toll signaling pathway of *Tenebrio molitor* (Coleoptera: Tenebrionidae) and other invertebrates (Park et al. 2011). In the overwintering *H. cunea* (Lepidoptera: Erebidae), the induction of serpin-serine protease pairs was discovered without rational deduction (Deng et al. 2018). Overall, the highly active immune system mediated by serine protease as a vital weapon of defense against pathogens and parasites is a typical inner attribute in the overwintering *M. alternatus* larvae.

Most insects that allow for their fitness costs have evolved a strategy to cope with annual cold winters (Bale and Hayward 2010, Sinclair 2015). Overwintering behavior was involved in a series of intrinsically regulated physiological processes in vivo. Based on our latest data on RNA-seq in the overwintering larvae, we acquired a more comprehensive understanding of this process in *M. alternatus*. It was implied that a successful overwintering process relied largely on the energy allocation trade-off of *M. alternatus*. The genes involved in energy-intensive physiological activities, such detoxification, molting, and transportation of trehalose, were inhibited, whereas a large number of functional genes involved in the synthesis of antifreeze compounds and innate immune were favored. Additionally, the elevation of PBAN and enrichment of cellular process confirmed the previous overwintering strategy of *M. alternatus*. Finally, our current understanding of overwintering behavior in *M. alternatus* remains largely unclear, and future studies warrant intensive exploration of the role of target genes at the molecular level.

## Supplementary Material

ieac025_suppl_Supplementary_Material

## Data Availability

Publicly available datasets were analyzed in this study. These data can be found here: https://www.ncbi.nlm.nih.gov/bioproject/?term=prjna645719. The gene sequences of Table 5 have been deposited on Genbank database with accession numbers: OM349508 to OM349534.
