# Supplementary material for: Comparative Transcriptome Analysis Reveals Molecular Insights in Overwintering Monochamus alternatus (Coleoptera: Cerambycidae)
Source: J Insect Sci. 2022 May 13;22(3):8. doi: 10.1093/jisesa/ieac025 (PMC9105011; doi:10.1093/jisesa/ieac025)
Supplement: ieac025_suppl_Supplementary_Material [file ieac025_suppl_supplementary_material.doc]

**Table S1. Oligonucleotide Primers Used for qRT-PCR validation.**

| Primer name | Primer sequence (5’→3’) | Amplification efficiencies R2 |
| --- | --- | --- |
| DN13641F  DN13641R  DN14927F  DN14927R  DN12538F  DN12538R  DN9141F  DN9141R  DN9405F  DN9405R  DN11915F  DN11915R  DN15899F  DN15899R  DN8217F  DN8217R  DN8907F  DN8907R  DN295F  DN295R  DN9677F  DN9677R  DN13804F  DN13804R  DN13783F  DN13783R | TGGCTGTAGCAGTACCACAA  CTGCTACAACAGCACCTTCC  TGGGCTGTCATGGGAGAAAT  TGCAGCAGACACCAAACATC  TCGAGCCATTGGTTCTTCCT  TCTGAACTGCTGCACGTCTA  ACGTCCAACACTTCAAACCG  AGTCTGCTACCATCGCAGTT  GTTGGACCCAGCCGATTTAC  ATAACCAGCAGGACAACGGA CGGTGACAACTGTGCAAGAA  GTCCTGTGCAAACGCAAGTA  GGTCACTACGCTTCACCAAC  CGTCAGCAGTTTCGTAAGCA  GCCCAGTGGGAGGAACTATT  GCCGCTCATCATTGGAACTT  GCAGCGAATGAGAAGCTGAA  TCCTTGGCACTGTCGTACTT  ATACAGTTGGCCACACCACT  CAGGACTTCCGGCACAGTAT  AGGTTTCCAACTTCCAACGC  CTTCCAGAAGACCCTCGGTT  CAAGTGGAGACCTGGTCGTA  TTCCCTGTCGTCAAATCCCA  CTCATATCCAGGCCCGAACT  GAACGGGTTGCGAGATTTCA | 0.95 0.992  1.02 0.995  0.98 0.997  0.97 0.992  1.01 0.993  1.05 0.997  0.94 0.999  1.03 0.998  1.01 0.995  0.99 0.997  0.94 0.999  0.98 0.996  1.10 0.997 |

**
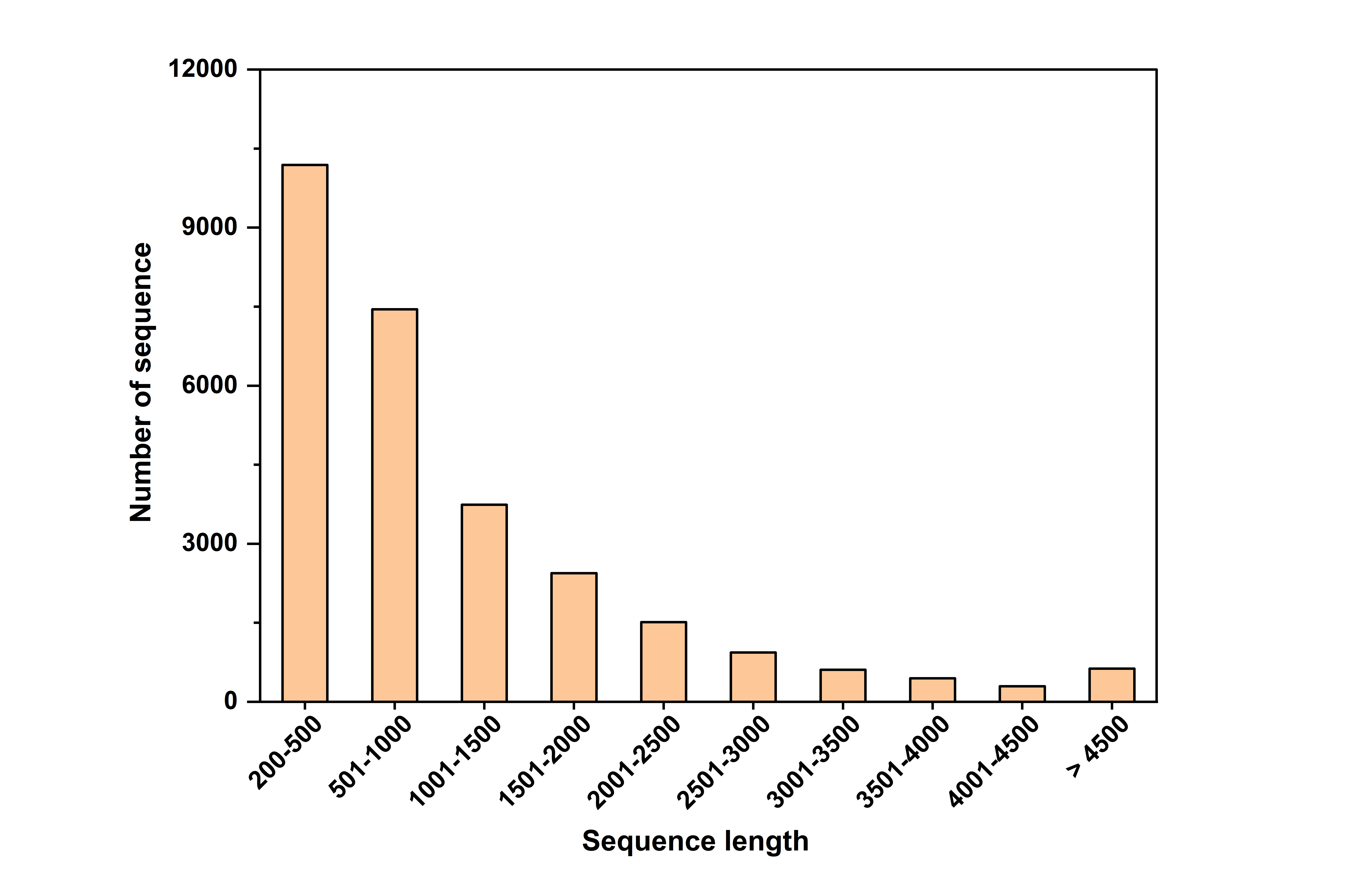
**

**Figure S1.** Length and number distribution of unigenes from *M. alternatus* non-overwintering overwintering larvae transcriptomes .

**
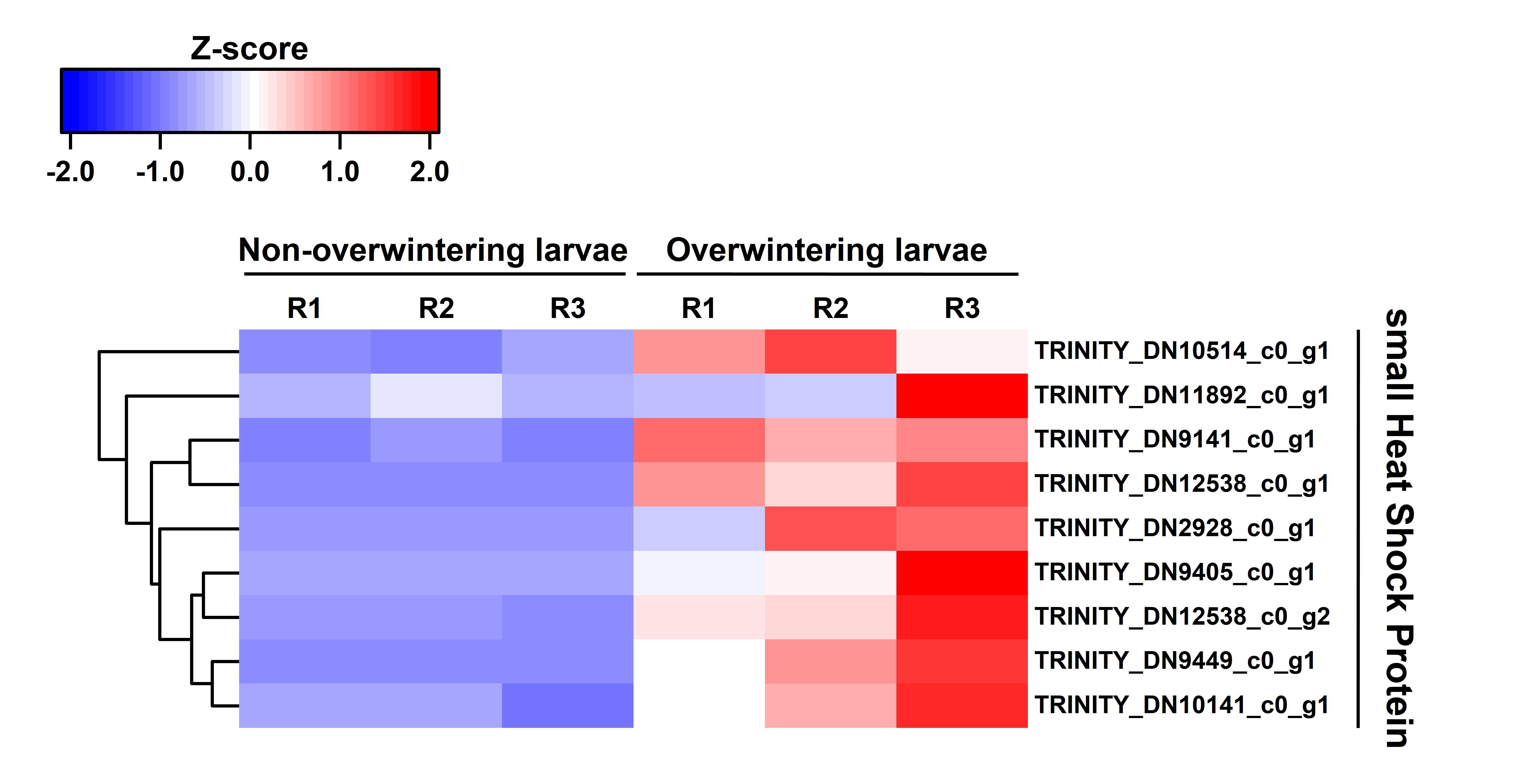
**

**Figure S2.** Heatmap and cluster analysis of DEGs encoding small Heat Shock Protein in non-overwintering larvae and overwintering larvae. The color scale bar showed expression levels after Z-score row normalization. Red bar indicated up-regulated while Blue bar indicated down-regulated. R1-3 represented three replicates.


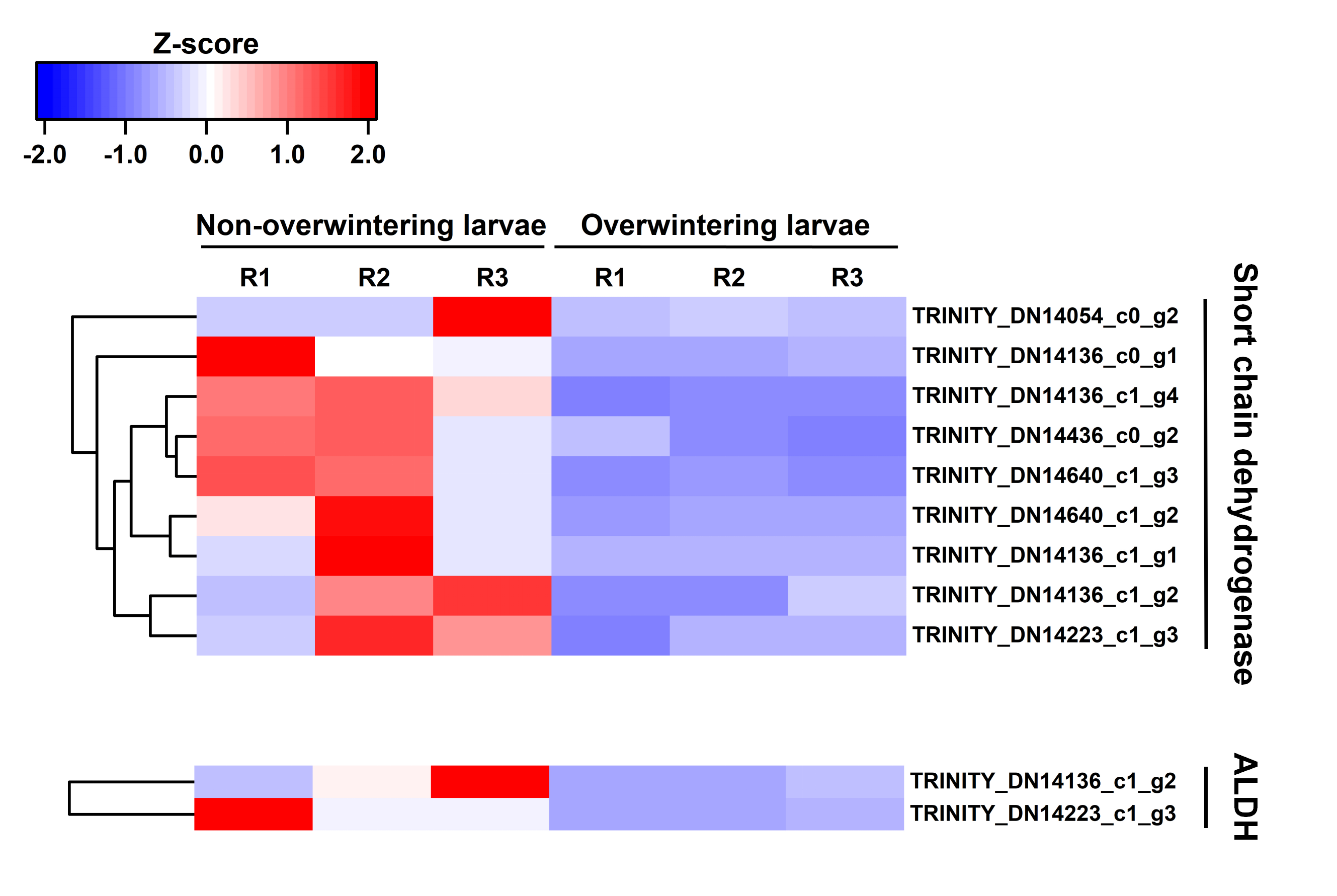


**Figure S3.** Heatmap and cluster analysis of DEGs encoding Short chain dehydrogenase and Aldehyde dehydrogenase (ALDH) in non-overwintering larvae and overwintering larvae. The color scale bar showed expression levels after Z-score row normalization. Red bar indicated up-regulated while Blue bar indicated down-regulated. R1-3 represented three replicates.
